# Evaluation of two new highly multiplexed PCR assays as an alternative to next‐generation sequencing for *IDH1/2* mutation detection

**DOI:** 10.1002/1878-0261.13311

**Published:** 2022-10-17

**Authors:** Loetitia Favre, Nouhoum Sako, Sihem Tarfi, Violaine Tran Quang, Corine Joy, Aurélie Dupuy, Erell Guillerm, Philippe Gaulard, Orianne Wagner‐Ballon, Anaïs Pujals, Ivan Sloma

**Affiliations:** ^1^ Department of Pathology AP‐HP, Henri Mondor University Hospital Creteil France; ^2^ Univ Paris Est Creteil, INSERM, IMRB France; ^3^ Hematology and Immunology Department AP‐HP, Henri Mondor University Hospital Creteil France; ^4^ Genetic Department AP‐HP, University Hospital Pitié Salpêtrière Paris France

**Keywords:** cancer, ddPCR, IDH1/2, leukemia, NGS, qPCR

## Abstract

*IDH1* and *IDH2* somatic mutations have been identified in solid tumors and blood malignancies. The development of inhibitors of mutant IDH1 and IDH2 in the past few years has prompted the development of a fast and sensitive assay to detect *IDH1* ^*R132*^, *IDH2* ^*R140*^ and *IDH2* ^*R172*^ mutations to identify patients eligible for these targeted therapies. This study aimed to compare two new multiplexed PCR assays – an automated quantitative PCR (qPCR) on the PGX platform and a droplet digital PCR (ddPCR) with next‐generation sequencing (NGS) for *IDH1/2* mutation detection. These assays were evaluated on 102 DNA extracted from patient peripheral blood, bone marrow and formalin‐fixed paraffin‐embedded tissue samples with mutation allelic frequency ranging from 0.6% to 45.6%. The ddPCR assay had better analytical performances than the PGX assay with 100% specificity, 100% sensitivity and a detection limit down to 0.5% on *IDH1* ^*R132*^, *IDH2* ^*R140*^ and *IDH2* ^*R172*^ codons, and a high correlation with NGS results. Therefore, the new highly multiplexed ddPCR is a fast and cost‐effective assay that meets most clinical needs to identify and follow cancer patients in the era of anti‐IDH1/2‐targeted therapies.

## Graphical abstract

This study presents the first evaluation of two new highly multiplexed PCR assays as compared with next‐generation sequencing (NGS): an automated qPCR on the PGX platform and an allele‐specific droplet digital PCR (ddPCR) assay, both designed to detect most *IDH1* and *IDH2* hotspot mutations. The ddPCR assay was the best assay in terms of sensitivity, specificity, robustness, cost and timeliness, regardless of patient sample processing. It is, therefore, an appealing alternative to NGS to screen patients eligible for IDH1/2 targeted therapies.

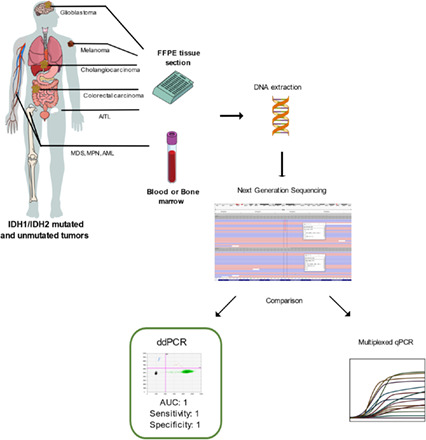

**Abbreviations**
2HG: (D)‐2‐Hydroxyglutarate
α‐KG: α‐ketoglutarate
AITL: angioimmunoblastic T‐cell lymphoma
AML: acute myeloid leukemia
CCA: cholangiocarcinomas
CRC: colorectal carcinoma
DNA: deoxyribonucleic acid
ddPCR: droplet digital PCR
FFPE: formalin‐fixed paraffin‐embedded
GB: glioblastomas
IDH1&2: Isocitrate Deshydrogenase 1&2
IGV: integrative genomics viewer
LOB: limit of blank
LOD: limit of detection
MDS: myelodysplastic syndromes
MPN: myeloproliferative neoplasms
NGS: next‐generation sequencing
qPCR: quantitative PCR
ROC: receiving operating characteristics
SNVs: single‐nucleotide variants
VAF: variant allele frequency

## 1 Introduction

Isocitrate dehydrogenase enzymes 1 (IDH1) and 2 (IDH2) form homodimers to catalyze the reversible NAD^+^ dependent oxidative decarboxylation of isocitrate to α‐ketoglutarate (α‐KG) [1]. IDH1 is localized in peroxisomes and the cytosol, whereas IDH2 is located in the mitochondria [2]. Since the discovery of a somatic *IDH1* mutation in colorectal cancer patients [3], other mutations of *IDH1* and IDH2 have been identified in many other cancers such as secondary glioblastoma and glioma [4, 5, 6] (~ 80%), chondrosarcoma (50–80%) [5, 7], thyroid carcinoma (16%) [5, 8] intrahepatic cholangiocarcinoma (10–20%) [5, 9], and in other types of solid tumors at lower frequencies [5, 10, 11]. *IDH1/2* mutations are also present in hematological malignancies, mainly in acute myeloid leukemia (AML, ~ 20%) [5, 12], but also in myeloproliferative neoplasms (MPN, ~ 1–4%) [13], in myelodysplastic syndromes (MDS, ~ 5%) [12] and angioimmunoblastic T‐cell lymphomas (AITL, ~ 20–30%) [5, 14]. *IDH1* and two mutations are missense mutations located on three hotspot codons: *IDH1* ^*R132*^, *IDH2* ^*R140*^, and *IDH2* ^*R172*^. These amino acids are localized within the catalytic domain of the enzymes. Their mutation results in the activation of a neomorphic activity that converts α‐KG into (D)‐2‐Hydroxyglutarate (2HG). The latter is an oncometabolite involved in DNA and histone hypermethylation leading to gene transcription deregulation and cell differentiation blockage [15]. The frequency of mutated codons varies across malignancies [5]. *IDH1* ^*R132*^ and *IDH2* ^*R172*^ are found in solid tumors, while *IDH2* ^*R140*^ is rarely mutated. On the contrary, all three *IDH1/2* codons can be altered in myeloid malignancies, and only *IDH2* ^*R172*^ is affected in AITL [16, 17].

In the past few years, anti‐IDH1 or IDH2 targeted therapies have been actively evaluated alone or in association with 5‐azacytidine in refractory, relapsed or newly diagnosed AML [18, 19, 20]. Several clinical trials are also evaluating these targeted therapies on advanced or refractory cholangiocarcinoma [21, 22], on advanced‐stage chondrosarcoma [23], or glioma [24]. The Food and Drug Administration (FDA) recently approved ivosidenib to treat newly diagnosed or relapsed AML [25], and for locally advanced or metastatic cholangiocarcinoma.

Given all these therapeutic developments, it has become increasingly necessary to genotype *IDH1* and *IDH2* hotspot codons for these malignancies. We and others have developed multiple PCR, immunohistochemistry [26, 27, 28, 29], mass spectrometry [7] or Sanger sequencing assays. However, most of these methods were not designed to detect mutations on all three codons with high specificity and sensitivity. For these reasons, next‐generation sequencing (NGS) has become the standard for *IDH1/2* mutation detection and quantification in many laboratories analyzing both solid tumors and hematological malignancies. However, NGS remains a cost and time‐consuming method that hardly meets the clinical need of fast result release compared to PCR assays. The objective of our study was to compare two new multiplexed PCR assays – an automated qPCR and a droplet digital PCR (ddPCR) as an alternative to NGS for *IDH1/2* mutation detection.

## 2 Materials and methods

### 2.1 Samples

**Fig. 1:**
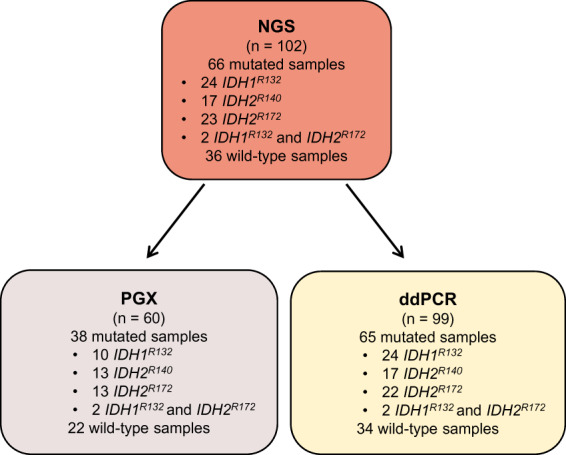
Description of DNA sample set used for ddPCR and PGX comparison with NGS. DNA samples from different *IDH1* ^*R132*^, *IDH2* ^*R140*^ or *IDH2* ^*R172*^ mutated and unmutated‐tumors were analyzed by NGS (*n* = 102), by PGX (*n* = 60) and ddPCR (*n* = 99). ddPCR, Droplet digital PCR; NGS, Next‐generation sequencing.

DNA samples from 102 patients with acute myeloid leukemia (AML), myelodysplastic syndromes (MDS), myeloproliferative neoplasms (MPN), angioimmunoblastic T‐cell lymphoma (AITL), cholangiocarcinomas (CCA), glioblastomas (GB), melanoma, colorectal carcinoma (CRC) or commercial control (HD829, Myeloid DNA Reference Standard, Horizon) were collected for this study and were assessed using NGS. This study was conducted as part of the care in Henri Mondor University Hospital in compliance with French regulations and approved (no. 2020‐083) by the Henri Mondor Institutional Review Board (No. 00011558). The study methodologies conformed to the standards set by the Declaration of Helsinki. All patient data were anonymized and deidentified before analysis. A letter of nonobjection explaining the purpose of this study was sent to each patient. Sixty DNA samples were assessed using Easy‐PGX‐ready IDH1/2 kit. Ninety‐nine DNA samples were evaluated using ddPCR (Table 1, Fig. 1, and Table S1).

**Table 1:** Sample set. AITL, angioimmunoblastic T‐cell lymphoma; AML, acute myeloid leukemia; CCA, cholangiocarcinomas; CRC, colorectal carcinoma; DNA, deoxyribonucleic acid; FFPE, formalin‐fixed paraffin‐embedded; GB, glioblastomas; MDS, myelodysplastic syndrome; MPN, myeloproliferative neoplasm; VAF, variant allele frequency; WT, wild‐type.

	Reference NGS	easypgx	ddPCR
	*n* [VAF Range]	*n*	*n* [VAF Range]
Pathology			
AML	20	7	20
MDS or MPN	37	31	37
AITL	26	21	23
CCA	13		13
GB	3		3
CRC	1		1
Melanoma	1		1
Commercial control (HD829)	1	1	1
Total	102	60	99
Samples			
Blood or bone marrow	57	38	57
FFPE	44	21	41
Commercial DNA (HD829)	1	1	1
Mutational status analysis			
WT samples			
*IDH1* ^*R132*^	76	48	73
*IDH2* ^*R140*^	85	49	82
*IDH2* ^*R172*^	77	55	75
*IDH1* ^*R132*^, *IDH2* ^*R140*^, *IDH2* ^*R172*^	36	22	34
Mutated samples			
*IDH1* ^*R132*^	26 [1–44]	12	26 [1–43.4]
*IDH2* ^*R140*^	17 [2.7–45.5]	11	17 [1.8–45.6]
*IDH2* ^*R172*^	25 [0.6–45]	5	24 [0.6–43.2]

### 2.2 DNA extraction

DNA extractions were performed from formalin‐fixed, paraffin‐embedded (FFPE) tissue sections (usually seven sections, 5 μm thick) using the Maxwell RSC DNA FFPE Kit IVD (Promega, Charbonnières‐Les‐Bains, France) as previously described [30, 31, 32], or from bone marrow and whole blood samples using QIAsymphony DSP DNA midi kit^®^ and QIsymphony extraction platform (Qiagen, Courtaboeuf, France) according to the manufacturer's instructions. DNAs were quantified using a Qubit fluorimeter with the Qubit dsDNA HS Array Kit (ThermoFisher Scientific, Ilkirch, France).

### 2.3 *IDH1/2* genotyping by next‐generation sequencing

DNA samples were sequenced using a multiplex PCR‐based enrichment (Ampliseq custom panel, ThermoFisher Scientific) or a custom hybridization capture‐based target enrichment (SureSelect QXT Library Prep Kit, Agilent, Courtaboeuf, France).

As previously described, [28], 10 ng of DNA was amplified using an Ampliseq custom panel (ThermoFisher Scientific), a multiplex PCR‐based library‐preparation method by which many regions (70–150 bp), including those coding for *IDH1* and *IDH2*, were amplified. Amplicons were then digested, barcoded and amplified using the Ion Ampliseq kit for Chef DL8 and Ion Xpress barcode adapter kit (ThermoFisher Scientific), according to the manufacturer's instructions. After DNA library preparation and quantification, 25 pm of each library was multiplexed and clonally amplified on ion‐sphere particles (ISP) by emulsion PCR performed on Ion Chef (ThermoFisher Scientific), according to the manufacturer's instructions. The ISP templates were loaded onto an Ion‐510 or 520 chip and sequenced on an S5 sequencer with the Ion 510™ & Ion 520™ & Ion 530™ Kit–Chef, according to the manufacturer's instructions. Run performance was assessed and data analyzed with the torrent suite Software v.5.10.0 (ThermoFisher Scientific). Sequencing data were analyzed using two different pipelines: ionreporter (ThermoFisher Scientific) and SeqPilot (JSI Medical Systems, Ettenheim, Germany). Single‐nucleotide variants (SNV) were visualized using the Variant Caller plug‐in version 5.10.0.18 with low stringency settings (threshold: 2%).

For the QXT assay, libraries were prepared according to the manufacturer's instructions, except that 70 ng of input DNA was used to prepare all libraries. 85 kb regions, including hotspots or all exons of 38 genes, were captured and sequenced with an Illumina v3 2 × 300 bp chip on MiSeq (Illumina^®^, San Diego, CA, USA). Demultiplexing and adapter trimming was performed with MiSeq reporter. Fastq files were trimmed with sickle (v1.33) to remove low‐quality bases and then aligned on hg19 reference genome with bwa‐mem (v0.7.15). Realignment and base score recalibration was performed with gatk (v3.8.0) according to the Broad Institute guidelines. SNVs and small indels were called with varscan2 (v2.4.3) and haplotype caller (v3.8.0) on the whole captured genomic positions. On specific hot spots (including *IDH1* ^*R132*^, *IDH2* ^*R140*^, and *IDH2* ^*R172*^), a second calling was performed with varscan2 using the following parameters: minimum supporting reads = 5; minimum base quality at a position to count a read = 13 and minimum variant allele frequency (VAF) threshold = 0.005 and Ignore variants with > 90% support on one strand = No. The Integrative Genomics Viewer (igv v 5.01; Broad Institute, Cambridge, MA, USA) was used for visual low vaf mutation curation.

### 2.4 *IDH1/2* genotyping by easypgx

DNA samples were assessed for *IDH1* (codons 105 and 132) and *IDH2* mutations (codons 140 and 172) using easypgx ^®^ ready *IDH1/2* kit (Diatech Pharmacogenetics, Jesi, Italy; Table S2).

Twenty‐five nanogram of DNA extracted from blood or FFPE is added for each position, and PCR is achieved according to the manufacturer's instructions. Data were analyzed using easypgx software.

### 2.5 *IDH1/2* genotyping by droplet digital PCR

An allele‐specific droplet digital PCR assay (Biorad^®^, Marnes‐la‐Coquette, France) designed to detect six mutations on *IDH1* ^*R132*^ codon (dHsaEXD61571942), four mutations on *IDH2* ^*R140*^ codon (dHsaEXD35841715) and five mutations on *IDH2* ^*R172*^ codon (dHsaEXD10111488) was evaluated (Table S3). Briefly, 1.1 μL of DNA at 30 or 50 ng·μL^−1^ was added to each master mix and then automatically encapsulated in 15 000 to 20 000 droplets. Fluorescent droplets were then quantified using the QX200 droplet reader (Biorad^®^). Each run was analyzed using quantasoft software (version 1.7.4; Biorad^®^). Positivity thresholds were set for each sample as the signal to background ratios depended on the codon and the type of mutation detected. When only double‐positive droplets (for mutant and wild‐type allele) were present, usually at a limited number (< 5 droplets) and displaying an intermediate fluorescence intensity, these were considered as negative artifactual events. In contrast, single positive droplets were always considered as positive events. Thresholds were thus adjusted accordingly. Following a temperature gradient PCR experiment, the annealing temperature was set at 55 °C as it optimally separated positive droplets from the fluorescence background signal. The number of droplets was converted by quantasoft into copies of mutant or wild‐type alleles for each codon using Poisson statistics. Results were expressed using the fractional abundance provided by quantasoft ^®^ software (Biorad^®^) as follows: %VAF = 100 * *IDH*_mutant_ copies ÷ (*IDH*_mutant_ copies + *IDH*_wildtype_ copies).

The following formula was applied to evaluate the limit of blank (LOB): LoB = mean_blank_ + 1.645 (SD_blank_) [33] with SDblank corresponding to the standard deviation (SD) of background allelic frequencies measured on all wild type codons evaluated in this study (Table 1; *IDH1* ^R132^, *n* = 73 samples, *IDH2* ^R140^, *n* = 82 samples, and *IDH2* ^R172^, *n* = 75 samples). The limit of detection (LOD) was estimated by two different methods. For LOD_1_, 30 ng of seven mutated DNA samples including sample 9 (*IDH1* ^*R132S*^, 32.8%), sample 3 (*IDH1* ^*R132C*^, 31.5%), sample 12 (*IDH1* ^*R132G*^, 33.9%), sample 33 (*IDH2* ^*R140Q*^, 32.8%), sample 27 (*IDH2* ^*R140W*^ 43.8%), sample 49 (*IDH2* ^*R172K*^, 31.6%) and sample 57 (*IDH2* ^*R172G*^ 15.0%) were serially diluted at a 1 : 4 ratio in a wild‐type (WT) DNA (sample 93) to obtain standards with VAF ranging from 44.3% to 0.03% (Table S1). The following formula was used: LOD_1_ = LOB + 1.645 × SD_Low_ with SDlow corresponding to the average standard deviation of variant allelic frequencies found for the last two dilutions (VAF at ~ 0.5% and VAF at ~ 0.125%) [33]. For LOD_2_, the following formula was applied: LOD_2_ = Mean_Blank_ + 5xSD_blank_.

### 2.6 Statistical analysis and diagnostic values

All data were collected using excel software. graphpad prism software version 6.0 and medcalc statistical software version 12.7.5 (Ostend, Belgium) were used to perform correlation tests, Bland–Altman graphs [34] and Receiving Operating Characteristics (ROC) curves. Accuracy, positive (+LR) and negative likelihood ratio (−LR) were calculated using formulas described in [35]. As no false positive result was detected, +LR values could not be determined (Table S4).

## 3 Results

### 3.1 Validation set

In this study, the performances of two methods – easypgx (Diatech Pharmacogenetics) and a multiplexed droplet digital PCR (ddPCR) assay were compared to targeted NGS sequencing. We selected 102 DNAs initially sequenced by NGS to detect and quantify *IDH1* ^*R132*^, *IDH2* ^*R140*^, and *IDH2* ^*R172*^ mutations. The characteristics of all samples analyzed are summarized in Fig. 1, Table 1 and Table S1. Among these, 57 samples were extracted from peripheral blood (PB), or bone marrow (BM) derived from AML (*n* = 20) or MPN/MDS (*n* = 37) patients. Forty‐four DNAs were extracted from formalin‐fixed paraffin‐embedded (FFPE) tissue samples from T‐cell lymphoma (*n* = 26), cholangiocarcinoma (*n* = 13), glioblastoma (*n* = 3), colorectal carcinoma (*n* = 1) or melanoma (*n* = 1) patients, and one commercial DNA was also included. Among this DNA set, 36 were wild type (WT) on all three codons, and 66 DNAs were mutated according to NGS results. Twenty‐four samples carried a single‐nucleotide variant (SNV) on codon 132 (R132C/S/G/L/H), 17 on codon 140 (R140W/Q), and 23 on codon 172 (R172K/T/G/S/W). Two samples had concomitant R132 and R172 mutations: the commercial DNA (both with a variant allelic frequency at 5%) and one AML patient DNA with concomitant *IDH1* ^R132C^ and *IDH2* ^R172K^ mutations below the variant calling threshold but was visually confirmed in the bam file (Fig. S1A). Altogether, 68 mutations were detected (66 single mutations and two double mutations) with VAF ranging from 0.4% to 45.5%, and 76, 85, and 77 DNAs were considered WT on 132, 140, and 172 codons, respectively. To evaluate PGX and ddPCR assay performance, 60 samples were analyzed by both assays, 24 by ddPCR only and two by PGX only.

### 3.2 PCR limit of blank and limit of detection

**Fig. 2:**
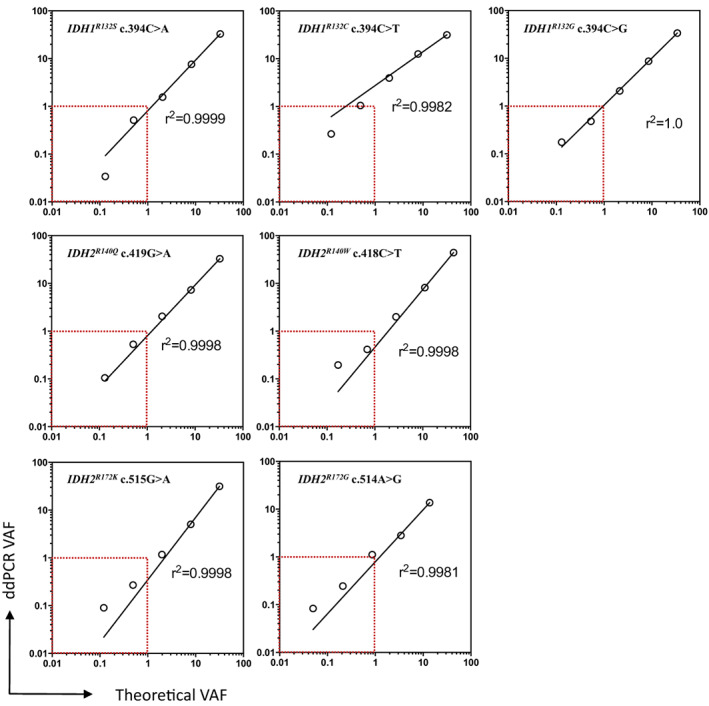
ddPCR limit of detection. The seven graphs represent the ddPCR variant allelic frequency (VAF) in terms of theoretical VAF obtained by multiplying *IDH1* (upper graphs) or *IDH2* (middle and lower panel) mutant VAF of the undiluted DNA sample by the dilution ratio. Each dot represents the mean of VAF quantified by ddPCR in duplicates, and the black lines correspond to the best fitting log–log line. *r* ^2^, Coefficient of determination; ddPCR, Droplet digital PCR.

PGX limit of detection (LOD) was determined and provided by PGX supplier at 0.5% for *IDH1* ^*R132S,R132G,R132L,R132I,R132V*^, 2% for *IDH1* ^*R132H,R132C*^ and *IDH2* ^*R140G,R140W,R140Q,R140W*^, 5% for *IDH2* ^*R172K*^ and 1% for *IDH1* ^*R172G,R172W,R172T,R172M,R172S*^ (Table S2). For ddPCR, the average background signal (mean_blank_) was evaluated on 73 WT samples for *IDH1* ^R132^, 82 for *IDH2* ^*R140*^, and 75 for *IDH2* ^*R172*^ and found statistically different between FFPE samples and non‐FFPE (blood/bone marrow) samples for *IDH1* ^*R132*^ (unpaired *t*‐test with Welch's correction, *P* = 0.0003), *IDH2* ^*R140*^ (unpaired *t*‐test with Welch's correction, *P* = 0.0001) and *IDH2* ^*R172*^ codons (unpaired *t*‐test with Welch's correction, *P* = 0.0091). The limit of blank (LOB) was found at 0.29% for codon *IDH1* ^*R132*^, 0.38% for codon *IDH2* ^*R140*^ and 0.11% for codon *IDH2* ^*R172*^ (Table 2). The LOB was further evaluated according to sample pre‐treatment. The LOB were lower for non‐FFPE samples (0.14% for codon *IDH1* ^*R132*^, 0.14% for codon *IDH2* ^*R140*^ and 0.02% for codon *IDH2* ^*R172*^) as compared with FFPE samples (0.44% for codon *IDH1* ^*R132*^, 0.51% for codon *IDH2* ^*R140*^ and 0.18% for codon *IDH2* ^*R172*^). The limit of detection (LOD) was assessed by serial dilution of seven DNA samples carrying *IDH1* ^*R132S*^, *IDH1* ^*R132*C^, *IDH1* ^*R132*G^, *IDH2* ^*R140Q*^, *IDH2* ^*R140W*^, *IDH2* ^*R172K*^, and *IDH2* ^*R172G*^ mutations mixed at a 1 : 4 ratio in a WT DNA to obtain standards with VAF ranging from 44.3% to 0.03% (Fig. 2). The coefficient of determination (*r* ^2^) ranged between 0.99 and 1 for the seven mutations analyzed, confirming the linearity of ddPCR on the entire range of VAF tested. However, for *IDH1* ^*R132C*^, *IDH2* ^*R140W*^, *IDH2* ^*R172G*^, and *IDH2* ^*R172K*^ mutations, ddPCR quantification was not linear when the VAF was below 1%. For all the mutations tested, ddPCR detected mutations with VAF down to 0.03%. The LOD was first calculated on all samples by two methods for each codon and ranged between 0.2% to 0.9% depending on the codon and the method of LOD calculation (Table 2 and Materials and methods). The LOD were also evaluated according to sample pre‐treatment and were found different. To take into account the significant increase in the ddPCR background signal associated with FFPE samples the LOD were finally set on all three codons at 0.5% and 1.2% for blood/bone marrow and FFPE samples respectively. These values corresponded to the highest LOD identified among the three codons analyzed.

**Table 2:** Estimation of ddPCR limit of blank and limit of detection. LOB, limit of blank; LOD, limit of detection. LOD_1_ = LOB + 1.645 × SD_low_ [33]. LOD_2_ = Mean_blank_ + 5 × SD_blank._ SD_low_, Standard deviation of variant allelic frequencies found by ddPCR between 0.03% and 1.09% following serial dilutions of seven DNA mutated on *IDH1* ^*R132*^, *IDH2* ^*R140*^ or *IDH2* ^*R172*^ codons (Fig. 2 and Materials and methods). FFPE, formalin‐fixed‐paraffin‐embedded.

	*IDH1* ^*R132*^	*IDH2* ^*R140*^	*IDH2* ^*R172*^
Mean_blank_ (%)	0.07	0.10	0.02
Mean_blank_ non FFPE (%)	0.02	0.02	0.004
Mean_blank_ FFPE (%)	0.16	0.18	0.05
SD_blank_	0.13	0.17	0.05
SD_blank_ non FFPE	0.1	0.1	0
SD_blank_ FFPE	0.2	0.2	0.1
LOB (%)	0.29	0.38	0.11
LOB non FFPE (%)	0.14	0.14	0.02
LOB FFPE (%)	0.44	0.51	0.18
LOD_1_ (%)	0.6	0.5	0.2
LOD_1_ non FFPE (%)	0.5	0.3	0.2
LOD_1_ FFPE (%)	0.8	0.7	0.3
LOD_2_ (%)	0.7	0.9	0.3
LOD_2_ non FFPE (%)	0.4	0.4	0.1
LOD_2_ FFPE (%)	1.0	1.2	0.5

### 3.3 Sensitivity and specificity of PGX and ddPCR

**Fig. 3:**
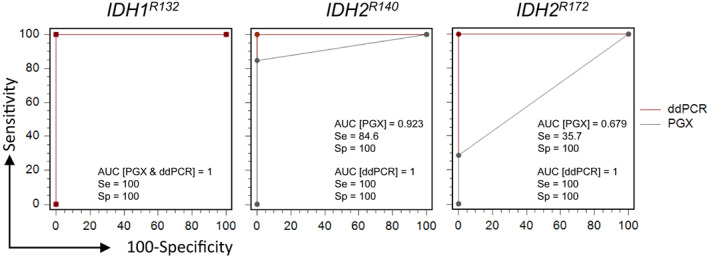
Sensitivity and specificity of multiplex PCR and ddPCR. Results for PGX (*n* = 60) and ddPCR (*n* = 99) are represented in gray and red lines, respectively, for the indicated *IDH1* (left panel) or *IDH2* (middle and right panel) codons. AUC, area under the curve; ddPCR, droplet digital PCR; Se, sensitivity; Sp, specificity.

All *IDH1* ^*R132*^ mutations were found by PGX and ddPCR (Table 1), leading to a sensitivity and specificity for both assays at 100% and an area under the curve (AUC) at 1 (Fig. 3).

PGX detected 11/13 *IDH2* ^*R140*^ mutations (Table 1 and Fig. 1). The two *IDH2* ^*R140Q*^ mutations found negative were detected at low allelic frequencies by NGS (4.8% and 6%), although the VAFs were above the PGX LOD (2%). All *IDH2* ^*R140*^ (*n* = 17) were found mutated by ddPCR (Table 1). Overall, the sensitivity for *IDH2* ^*R140*^ was 100% and 84.6% for ddPCR and PGX, respectively. AUC of ROC curves comparing both assays to NGS results was found at 1.0 and 0.923 for ddPCR and PGX, respectively (Fig. 3).

Only 5/14 *IDH2* ^*R172*^ mutations (NGS VAF = 3% to 42%) were detected by PGX (LOD = 5% and 1%, Table S1) leading to a sensitivity at 35.7%. Conversely, ddPCR detected 22/22 mutations (NGS VAF = 0.6% to 45%). ROC curve AUCs were found at 1 and 0.679 for ddPCR and PGX, respectively. There was no false‐positive result for both assays on all three codons (Fig. 3).

Sensitivities (Se), specificities (Sp), negative and positive predictive values (NPV and PPV), likelihood ratios (LR) and accuracies were calculated for each codon for PGX and ddPCR, on the whole sample set and according to sample pre‐treatment (Table S4). ddPCR was found equivalent and often superior to PGX for all these parameters. Notably, ddPCR negative LR were always found at 0, while it ranged between 0.15 and 0.67 for PGX.

Considering DNA samples tested on all three codons of *IDH1* and *IDH2*, ddPCR showed much better analytical performances with a sensitivity, specificity, accuracy, negative and positive predictive values on all three codons at 100% compared with the PGX assay showing a lower sensitivity at 73.7%. Altogether, these results strengthen the analytical performance of ddPCR assay as compared with PGX.

### 3.4 ddPCR correlation with NGS

**Fig. 4:**
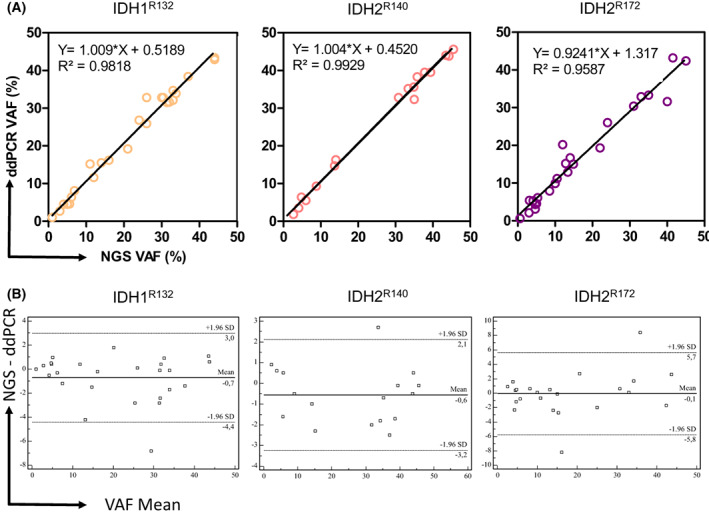
Correlation between NGS and ddPCR. (A) Correlation analysis between NGS variant allele frequency (VAF) and ddPCR VAF for *IDH1* ^*R132*^ (*n* = 26), *IDH2* ^*R140*^ (*n* = 17) and *IDH2* ^*R172*^ (*n* = 24). (B) Bland and Altman Diagrams for *IDH1* ^*R132*^ (*n* = 26), *IDH2* ^*R140*^ (*n* = 17) and *IDH2* ^*R172*^ (*n* = 24). The *x*‐axis corresponds to the average VAF between ddPCR and NGS for each sample. The *y*‐axis represents NGS_VAF_‐ddPCR_VAF_. ddPCR, droplet digital PCR.

The comparison of VAF found by NGS and ddPCR showed an excellent correlation for all mutations: *IDH1* ^*R132*^ (*r* ^2^ = 0.982, *P* < 0.0001, linear regression), *IDH2* ^*R140*^, (*r* ^2^ = 0.993; *P* < 0.0001; linear regression) and *IDH2* ^*R172*^ (*r* ^2^ = 0.959; *P* < 0.0001, linear regression; Fig. 4A). Although ddPCR showed an excellent correlation with NGS on the three assays, *r* ^2^ was lower for *IDH1* ^*R132*^ and *IDH2* ^*R172*^. 11/26 (42%) and 16/25 (64%) DNA samples carrying an *IDH1* ^*R132*^ *and IDH2* ^*R172*^ mutation were derived from FFPE samples. We, therefore, evaluated the correlation between ddPCR and NGS according to sample processing before DNA extraction. DNA samples derived from blood/marrow or FFPE samples showed a similar correlation with NGS results (for *IDH1* ^*R132*^, *r* ^2^ = 0.98 and *r* ^2^ = 0.95 and for *IDH2* ^*R172*^ *r* ^2^ = 0.97 and *r* ^2^ = 0.95 respectively; data not shown). Taken together, these results showed an excellent correlation between VAF obtained by NGS and ddPCR, including low VAF (under 1%) on peripheral blood, bone marrow, or FFPE samples.

The Bland and Altman diagrams did not identify a significant bias of VAF measured by ddPCR compared with NGS on *IDH1* ^*R132*^, *IDH2* ^*R140*^, and *IDH2* ^*R172*^ codons (mean bias = −0.7, −0.6 and 0.1 respectively), confirming a good agreement between both assays (Fig. 4B).

## 4 Discussion

The detection of *IDH1* ^*R132*^ and *IDH2* ^*R140/R172*^ mutations is helpful information to guide the diagnosis of several malignancies such as glioblastomas [4], gliomas [6], AITL [17], MDS [36], or AML [37] and identify patients eligible for anti‐IDH1 and/or anti‐IDH2 targeted therapies. Given the running time of NGS analysis, the implementation of another rapid, sensitive and specific assay would be a valuable improvement in the molecular characterization of cancer patients. In this study, two new multiplexed PCR methods were compared to NGS. The ddPCR assay was an excellent alternative to NGS for detecting and quantifying *IDH1* ^*R132*^, *IDH2* ^*R140*^ and *IDH2* ^*R172*^ mutations as it showed 100% specificity, 100% sensitivity, and a LOD estimated at 0.5% and 1.2% on *IDH1* ^*R132*^, *IDH2* ^*R140*^, and *IDH2* ^*R172*^ codons for blood/bone marrow samples and FFPE samples respectively. While the PGX PCR assay is not quantitative, *IDH1/2* mutant frequencies measured by ddPCR were linear between 1% and 45.6% and showed a high correlation with VAF obtained by NGS results. In contrast, the sensitivity of the automated multiplex PCR easy‐pgx was lower, thus limiting its potential for *IDH1/2* hotspot codon screening on clinical samples. Indeed, PGX failed to detect two mutations at codon 140, whose VAFs were 4.8% and 6%, while the supplier's LOD was 2%. Similarly, PGX failed to detect 10/15 *IDH2* ^*R172*^ mutations, including 5/8 *IDH2* ^*R172K*^ mutations with VAF up to 13%. Nevertheless, PGX identified the rare *IDH2* ^*R172T*^ and *IDH2* ^*R172W*^ mutations at 3.9% and 3.1% VAF, respectively (Table S1). Notably, most *IDH2* ^*R172*^ mutated samples genotyped by PGX (*n* = 11/15) were extracted from T‐cell lymphoma FFPE samples. Conversely, *IDH1* ^*R132*^ and *IDH2* ^*R140*^ mutated samples tested by PGX were extracted from blood or bone marrow samples. PGX sensitivity was higher on these samples at 100% and 84.4% for *IDH1* ^*R132*^ and *IDH2* ^*R140*^, respectively. These results suggest that PGX sensitivity may be significantly altered on FFPE samples. The PGX LOD was estimated using commercial DNA or recombinant plasmids that may not be a good surrogate of the patient's DNA, especially those obtained from FFPE tissues. These data underline the importance, for PGX assay, of re‐evaluating the LOD on patient samples for each of the mutations tested.

Finally, in hematological malignancies, the VAF can be low (< 5%), especially for patients' follow‐up after anti‐IDH therapy or in AITL patients [28]. In terms of sensitivity, ddPCR assay has proven to be the best method compared with PGX qPCR with a LOD estimated at 0.5% for blood/bone marrow samples and 1.2% for FFPE samples. However, it must be noted that the multiplexed ddPCR assay can detect a mutated codon but does not identify the exact mutation. In addition, this assay does not detect rare mutations such as *IDH2* ^*R172T*^. Nevertheless, according to the COSMIC (Catalogue Of Somatic Mutations In Cancer) database, this design allows the detection of 99.8% and 98.9% of the mutations identified for the *IDH1* and *IDH2* genes, respectively (Table S3).

## 5 Conclusions

The multiplexed ddPCR assay evaluated is a fast and quantitative method that significantly spares technical time and reduces analysis cost compared with NGS. It also covers most *IDH1/2* mutations with a LOD adapted to patient sample screening and therefore could meet most clinical needs of cancer molecular diagnostics laboratories.

## Conflict of interest

The authors declare no conflict of interest.

## Author contributions

AP and IS supervised and designed the experiments for PGX qPCR and ddPCR, respectively. NS, ST, CJ, and VTQ performed the experiments. LF, NS, ST, AP, EG, OW‐B, PG, AD and IS analyzed and interpreted data. LF, AP, and IS wrote the manuscript with OW‐B, PG, NS, AD, and ST inputs. All authors approved the manuscript.

## Supporting information


**Table S1.** Sample genotyping by NGS, PGX, and ddPCR.
**Table S2.** Mutations assessed by EasyPGX ready IDH1/2 kit.
**Table S3.** Mutations assessed by ddPCR.
**Table S4.** Diagnostic test values.

**Figure S1.** Detection of an additional mutation by ddPCR below the detection limit of NGS.

## Data Availability

The supporting data are available in the Table S1.
