# Supplementary material for: Evaluation of two new highly multiplexed PCR assays as an alternative to next‐generation sequencing for IDH1/2 mutation detection
Source: Mol Oncol. 2022 Oct 17;16(22):3916–26. doi: 10.1002/1878-0261.13311 (PMC9718115; doi:10.1002/1878-0261.13311)
Supplement: Supplementary file 1 — Table S1. Sample genotyping by NGS, PGX, and ddPCR. Table S2. Mutations assessed by EasyPGX ready IDH1/2 kit. Table S3. Mutations assessed by ddPCR. Table S4. Diagnostic test values. [file MOL2-16-3916-s001.docx]

**Supplementary information**

**Supplementary Figure S1.** Detection of an additional mutation by ddPCR below the detection limit of NGS.

(A) IGV Screenshots showing the R172 codon of IDH2 carrying a variant with R172K with an estimated variant allelic frequency at 0.4% (upper panel) and an example of a wild-type patient at the same position (lower panel). (B) Quantasoft^®^ dot plot showing the R172 mutant detected at 0.6% by ddPCR. Mutated droplets (blue), the double-positive droplets (orange), the wild-type droplets (green), and the empty droplets (grey) are depicted according to the quadrant thresholds (pink lines). ddPCR: droplet digital PCR. IGV : Integrative Genomics Viewer.

**Supplementary Table S1.** Sample genotyping by NGS, PGX, and ddPCR.

*IDH1^R132^*, *IDH2^R140,^* and *IDH2^R172^* codon mutations are colored in greyscale background. Wild-Type sample genotypes are in white background. VAF are indicated for NGS and ddPCR results. PGX results are indicated as MUT or NEG for mutation detection or mutation-negative, respectively. Sample #2 and #8 are double mutated DNAs from patient and commercial control HD829, respectively. * Delta Cq (dCq) is superior to 10. dCq = 12.1. HD829: Myeloid DNA Reference Standard (horizon^®^). FFPE : Formalin-Fixed Paraffin-Embedded; VAF: Variant Allele Frequency.

**Supplementary Table S2.** Mutations assessed by EasyPGX ready IDH1/2 kit.

**Supplementary Table S3.** Mutations assessed by ddPCR.

**Supplementary Table S4.** Diagnostic test values.

Sensitivity, Specificity, Negative Predictive Value, Positive Predictive Value, Positive likelihood ratio (+LR), Negative likelihood ratio (-LR), and accuracy were calculated according to the technique used, PGX and ddPCR, and according to sample types (FFPE samples versus blood/bone marrow samples) or for all samples. NA: Not applicable.

**Supplementary Table S1.** Sample genotyping by NGS, PGX, and ddPCR.

| **Sample #** | **Diagnosis** | **Pre-treatment** | **p.** | **c.** | **VAF NGS (%)** | **PGX** | **VAF ddPCR (%)** |
| --- | --- | --- | --- | --- | --- | --- | --- |
| 1 | MPN/MDS |  | IDH1 R132C | c.394C>T | 5.6 |  | 4.6 |
| 2 | MPN/MDS |  | IDH1 R132C | c.394C>T | 6.9 | MUT | 8.1 |
|  |  |  | IDH2 R172K | c.515G>A | 0.4 | **NEG*** | 0.6 |
| 3 | MPN/MDS |  | IDH1 R132C | c.394C>T | 31.4 |  | 31.5 |
| 4 | AML |  | IDH1 R132C | c.394C>T | 12.0 | MUT | 11.6 |
| 5 | AML |  | IDH1 R132C | c.394C>T | 5.0 |  | 4.6 |
| 6 | AML |  | IDH1 R132C | c.394C>T | 1.0 | MUT | 1.0 |
| 7 | AML |  | IDH1 R132C | c.394C>T | 26.0 | MUT | 25.9 |
| 8 | Commercial | HD829 | IDH1 R132C | c.394C>T | 5.0 | MUT | 4.5 |
|  |  |  | IDH2 R172K | c.515G>A | 5.0 | **NEG** | 4.5 |
| 9 | MPN/MDS |  | IDH1 R132S | c.394C>A | 30.4 | MUT | 32.8 |
| 10 | AML |  | IDH1 R132S | c.394C>A | 44.0 | MUT | 43.4 |
| 11 | MPN/MDS |  | IDH1 R132S | c.394C>A | 33.0 | MUT | 34.7 |
| 12 | MPN/MDS |  | IDH1 R132G | c.394C>G | 33.8 | MUT | 33.9 |
| 13 | AML |  | IDH1 R132L | c.395G>T | 33.0 | MUT | 32.1 |
| 14 | AML |  | IDH1 R132H | c.395G>A | 3.0 | MUT | 2.7 |
| 15 | MPN/MDS |  | IDH1 R132H | c.395G>A | 6.1 | MUT | 6.4 |
| 16 | CCA | FFPE | IDH1 R132C | c.394C>T | 16 |  | 16.2 |
| 17 | CCA | FFPE | IDH1 R132C | c.394C>T | 24 |  | 26.8 |
| 18 | CRC | FFPE | IDH1 R132C | c.394C>T | 30 |  | 32.8 |
| 19 | CCA | FFPE | IDH1 R132C | c.394C>T | 37 |  | 38.4 |
| 20 | Melanoma | FFPE | IDH1 R132C | c.394C>T | 21 |  | 19.2 |
| 21 | CCA | FFPE | IDH1 R132G | c.394C>G | 14 |  | 15.5 |
| 22 | CCA | FFPE | IDH1 R132H | c.395G>A | 4 |  | 4.5 |
| 23 | GB | FFPE | IDH1 R132H | c.395G>A | 44 |  | 42.9 |
| 24 | CCA | FFPE | IDH1 R132I | c.395G>T | 11 |  | 15.2 |
| 25 | CCA | FFPE | IDH1 R132C | c.394C>T | 32 |  | 31.6 |
| 26 | CCA | FFPE | IDH1 R132C | c.394C>T | 26 |  | 32.8 |
| 27 | MPN/MDS |  | IDH2 R140W | c.418C>T | 44.3 |  | 43.8 |
| 28 | AML |  | IDH2 R140W | c.418C>T | 14.0 |  | 16.3 |
| 29 | MPN/MDS |  | IDH2 R140Q | c.419G>A | 4.8 | **NEG** | 6.4 |
| 30 | MPN/MDS |  | IDH2 R140Q | c.419G>A | 35.8 | MUT | 38.3 |
| 31 | MPN/MDS |  | IDH2 R140Q | c.419G>A | 4.1 | MUT | 3.5 |
| 32 | MPN/MDS |  | IDH2 R140Q | c.419G>A | 13.7 | MUT | 14.7 |
| 33 | MPN/MDS |  | IDH2 R140Q | c.419G>A | 30.8 | MUT | 32.8 |
| 34 | MPN/MDS |  | IDH2 R140Q | c.419G>A | 2.7 |  | 1.8 |
| 35 | MPN/MDS |  | IDH2 R140Q | c.419G>A | 33.4 | MUT | 35.2 |
| 36 | MPN/MDS |  | IDH2 R140Q | c.419G>A | 34.9 | MUT | 35.6 |
| 37 | MPN/MDS |  | IDH2 R140Q | c.419G>A | 37.8 | MUT | 39.5 |
| 38 | MPN/MDS |  | IDH2 R140Q | c.419G>A | 39.4 | MUT | 39.5 |
| 39 | MPN/MDS |  | IDH2 R140Q | c.419G>A | 43.5 | MUT | 44 |
| 40 | MPN/MDS |  | IDH2 R140Q | c.419G>A | 8.8 | MUT | 9.3 |
| 41 | MPN/MDS |  | IDH2 R140Q | c.419G>A | 45.5 | MUT | 45.6 |
| 42 | AML |  | IDH2 R140Q | c.419G>A | 6.0 | **NEG** | 5.5 |
| 43 | AML |  | IDH2 R140Q | c.419G>A | 35.0 |  | 32.3 |
| 44 | AML |  | IDH2 R172K | c.515G>A | 33.0 |  | 32.9 |
| 45 | AML |  | IDH2 R172K | c.515G>A | 45.0 |  | 42.4 |
| 46 | AML |  | IDH2 R172K | c.515G>A | 31.0 |  | 30.4 |
| 47 | AML |  | IDH2 R172K | c.515G>A | 22.0 |  | 19.3 |
| 48 | AML |  | IDH2 R172K | c.515G>A | 24.0 |  | 26.0 |
| 49 | AML |  | IDH2 R172K | c.515G>A | 40.0 |  | 31.6 |
| 50 | MPN/MDS |  | IDH2 R172K | c.515G>A | 4.7 | MUT | 4.3 |
| 51 | AITL | FFPE | IDH2 R172K | c.515G>A | 12.0 | MUT | 20.2 |
| 52 | AITL | FFPE | IDH2 R172K | c.515G>A | 10.0 | **NEG** | 9.9 |
| 53 | AITL | FFPE | IDH2 R172T | c.515G>C | 3.9 | MUT | NA |
| 54 | AITL | FFPE | IDH2 R172K | c.515G>A | 4.1 | **NEG*** | 5.3 |
| 55 | AITL | FFPE | IDH2 R172K | c.515G>A | 41.5 | MUT | 43.2 |
| 56 | AITL | FFPE | IDH2 R172K | c.515G>A | 13.4 | **NEG** | 12.9 |
| 57 | AITL | FFPE | IDH2 R172G | c.514A>G | 14.9 |  | 15.0 |
| 58 | AITL | FFPE | IDH2 R172G | c.514A>G | 4.7 |  | 3.1 |
| 59 | AITL | FFPE | IDH2 R172G | c.514A>G | 3.0 | **NEG** | 2.1 |
| 60 | AITL | FFPE | IDH2 R172G | c.514A>G | 10.5 | **NEG** | 11.2 |
| 61 | AITL | FFPE | IDH2 R172G | c.514A>G | 12.8 | **NEG** | 15.2 |
| 62 | AITL | FFPE | IDH2 R172S | c.515G>C | 5.3 | **NEG** | 6.1 |
| 63 | AITL | FFPE | IDH2 R172W | c.514A>T | 3.1 | MUT | 5.4 |
| 64 | AITL | FFPE | IDH2 R172S | c.516G>TT | 8.5 | **NEG** | 7.9 |
| 65 | CCA | FFPE | IDH2 R172W | c.514A>T | 35 |  | 33.3 |
| 66 | CCA | FFPE | IDH2 R172K | c.515G>A | 14 |  | 16.7 |
| 67 | AITL | FFPE | WT | WT | <2 | NEG | <1.2 |
| 68 | AITL | FFPE | WT | WT | <2 |  | <1.2 |
| 69 | AITL | FFPE | WT | WT | <2 |  | <1.2 |
| 70 | AITL | FFPE | WT | WT | <2 |  | <1.2 |
| 71 | AML |  | WT | WT | <0.6 |  | <0.5 |
| 72 | AML |  | WT | WT | <0.6 |  | <0.5 |
| 73 | AML |  | WT | WT | <0.6 |  | <0.5 |
| 74 | AML |  | WT | WT | <0.6 |  | <0.5 |
| 75 | MPN/MDS |  | WT | WT | <0.6 |  | <0.5 |
| 76 | MPN/MDS |  | WT | WT | <0.6 |  | <0.5 |
| 77 | MPN/MDS |  | WT | WT | <0.6 | NEG | <0.5 |
| 78 | MPN/MDS |  | WT | WT | <0.6 | NEG | <0.5 |
| 79 | MPN/MDS |  | WT | WT | <0.6 | NEG | <0.5 |
| 80 | AITL | FFPE | WT | WT | <2 | NEG | <1.2 |
| 81 | AITL | FFPE | WT | WT | <2 | NEG | <1.2 |
| 82 | AITL | FFPE | WT | WT | <2 | NEG | <1.2 |
| 83 | AITL | FFPE | WT | WT | <2 | NEG |  |
| 84 | AITL | FFPE | WT | WT | <2 | NEG | <1.2 |
| 85 | AITL | FFPE | WT | WT | <2 | NEG | <1.2 |
| 86 | AITL | FFPE | WT | WT | <2 | NEG |  |
| 87 | AITL | FFPE | WT | WT | <2 | NEG | <1.2 |
| 88 | MPN/MDS |  | WT | WT | <0.6 | NEG | <0.5 |
| 89 | MPN/MDS |  | WT | WT | <0.6 | NEG | <0.5 |
| 90 | MPN/MDS |  | WT | WT | <0.6 | NEG | <0.5 |
| 91 | MPN/MDS |  | WT | WT | <0.6 | NEG | <0.5 |
| 92 | MPN/MDS |  | WT | WT | <0.6 | NEG | <0.5 |
| 93 | MPN/MDS |  | WT | WT | <0.6 | NEG | <0.5 |
| 94 | MPN/MDS |  | WT | WT | <0.6 | NEG | <0.5 |
| 95 | MPN/MDS |  | WT | WT | <0.6 | NEG | <0.5 |
| 96 | MPN/MDS |  | WT | WT | <0.6 | NEG | <0.5 |
| 97 | MPN/MDS |  | WT | WT | <0.6 | NEG | <0.5 |
| 98 | GB | FFPE | WT | WT | <2 |  | <1.2 |
| 99 | GB | FFPE | WT | WT | <2 |  | <1.2 |
| 100 | CCA | FFPE | WT | WT | <2 |  | <1.2 |
| 101 | CCA | FFPE | WT | WT | <2 |  | <1.2 |
| 102 | CCA | FFPE | WT | WT | <2 |  | <1.2 |

AML: Acute myeloid leukemia. AITL Angioimmunoblastic T-cell lymphoma. CCA: Cholangiocarcinoma. CRC: Colorectal carcinoma. GB Glioblastoma. FFPE: Formalin-fixed paraffin-embedded. MPN/MDS: Myeloproliferative neoplasm or myelodysplastic syndrome. MUT: mutated. NEG: Unmutated. **NEG**: False negative result. WT: Wild-type. * Not considered as false negative as the VAF is under detection threshold for PGX

**Supplementary Table S2.** Mutations assessed by EasyPGX ready IDH1/2 kit.

|  | **Well number** |  | **Variant name** | **CDS Mutation** | **LOD (%)** |
| --- | --- | --- | --- | --- | --- |
| **IDH1 codon 132** | 1 |  | G105G | c.315C>T | 2 |
|  | 2 |  | R132H | c.395G>A | 2 |
|  | 3 |  | R132C | c.394C>T | 2 |
|  | 4 |  | R132S* | c.394C>A | 0,5 |
|  |  |  | R132G* | c.394C>G |  |
|  |  |  | R132L* | c.395G>T |  |
|  |  |  | R132I* | c.394_395delinsAT |  |
|  |  |  | R132V* | c.394_395delinsGT |  |
| **IDH2**  **codon 140** | 5 |  | R140G* | c.418C>G | 2 |
|  |  |  | R140W* | c.418C>T |  |
|  |  |  | R140Q* | c.419G>A |  |
|  |  |  | R140L* | c.419G>T |  |
| **IDH2**  **codon 172** | 6 |  | R172K | c.515G>A | 5 |
|  | 7 |  | R172G* | c.514A>G | 1 |
|  |  |  | R172W* | c.514A>T |  |
|  |  |  | R172T* | c.515G>C |  |
|  |  |  | R172M* | c.515G>T |  |
|  |  |  | R172S* | c.516G>C or G>T |  |

LOD : Limit of Detection

* Indistinguishable mutations

**Supplementary Table S3.** Mutations assessed by ddPCR.

| Variant Name | CDS Mutation | COSMIC ID | Well number | Fluorophore |
| --- | --- | --- | --- | --- |
| *IDH1^R132C^* | c.394C>T | COSM28747 | 1 | FAM |
| *IDH1^R132L^* | c.395G>T | COSM28750 | 1 | FAM |
| *IDH1^R132S^* | c.394C>A | COSM28748 | 1 | FAM |
| *IDH1^R132G^* | c.394C>G | COSM28749 | 1 | FAM |
| *IDH1^R132H^* | c.395G>A | COSM28746 | 1 | FAM |
| *IDH1^R132V^* | c.394_395CG>GT | COSM28751 | 1 | FAM |
| *IDH1^WT^* | N/A | N/A | 1 | HEX |

| Variant Name | CDS Mutation | COSMIC ID | Well number | Fluorophore |
| --- | --- | --- | --- | --- |
| *IDH2^R140L^* | c.419G>T | COSM41875 | 2 | FAM |
| *IDH2*^R140W^ | c.418C>T | COSM41877 | 2 | FAM |
| *IDH2^R140Q^* | c.419G>A | COSM41590 | 2 | FAM |
| *IDH2 WT* | N/A | N/A | 2 | HEX |

| Variant Name | CDS Mutation | COSMIC ID | Well number | Fluorophore |
| --- | --- | --- | --- | --- |
| *IDH2^R172S^* | c.516G>T | COSM34090 | 3 | FAM |
| *IDH2^R172S^* | c.516G>C | COSM133672 | 3 | FAM |
| *IDH2^R172W^* | c.514A>T | COSM34039 | 3 | FAM |
| *IDH2^R172M^* | c.515G>T | COSM33732 | 3 | FAM |
| *IDH2^R172K^* | c.515G>A | COSM33733 | 3 | FAM |
| *IDH2^R172G^* | c.514A>G | COSM33731 | 3 | FAM |
| *IDH2^WT^* | N/A | N/A | 3 | HEX |

CDS : Coding sequence. N/A : not applicable. WT: wild-type

**Supplementary Table S4**. Diagnostic test values.

|  |  | PGX | | | ddPCR | | |
| --- | --- | --- | --- | --- | --- | --- | --- |
|  |  | R132 | R140 | R172 | R132 | R140 | R172 |
| FFPE samples | Sensitivity | NA | NA | 0.4 | 1 | NA | 1 |
|  | Specificity | NA | NA | 1 | 1 | NA | 1 |
|  | PPV | NA | NA | 1 | 1 | NA | 1 |
|  | NPV | NA | NA | 0.64 | 1 | NA | 1 |
|  | +LR | NA | NA | NA | NA | NA | NA |
|  | -LR | NA | NA | 0.67 | 0 | NA | 0 |
|  | Accuracy | NA | NA | 0.71 | 1 | NA | 1 |
| Blood/ bone marrow samples | Sensitivity | 1 | 0.84 | 0.5 | 1 | 1 | 1 |
|  | Specificity | 1 | 1 | 1 | 1 | 1 | 1 |
|  | PPV | 1 | 1 | 1 | 1 | 1 | 1 |
|  | NPV | 1 | 0.93 | 0.97 | 1 | 1 | 1 |
|  | +LR | NA | NA | NA | NA | NA | NA |
|  | -LR | 0 | 0.15 | 0.5 | 0 | 0 | 0 |
|  | Accuracy | 1 | 0.95 | 0.97 | 1 | 1 | 1 |
| FFPE + blood/bone marrow samples | Sensitivity | 1 | 0.84 | 0.36 | 1 | 1 | 1 |
|  | Specificity | 1 | 1 | 1 | 1 | 1 | 1 |
|  | PPV | 1 | 1 | 1 | 1 | 1 | 1 |
|  | NPV | 1 | 0.96 | 0.84 | 1 | 1 | 1 |
|  | +LR | NA | NA | NA | NA | NA | NA |
|  | -LR | 0 | 0.15 | 0.64 | 0 | 0 | 0 |
|  | Accuracy | 1 | 0.97 | 0.85 | 1 | 1 | 1 |

+LR: positive likelihood ratio. –LR: negative likelihood ratio. PPV: Positive predictive value. NPV: Negative predictive value. NA: Not Applicable.
