# Supplementary figures and images for: Evaluation of two new highly multiplexed PCR assays as an alternative to next‐generation sequencing for IDH1/2 mutation detection
Source: Mol Oncol. 2022 Oct 17;16(22):3916–26. doi: 10.1002/1878-0261.13311 (PMC9718115; doi:10.1002/1878-0261.13311)

## Slide 1
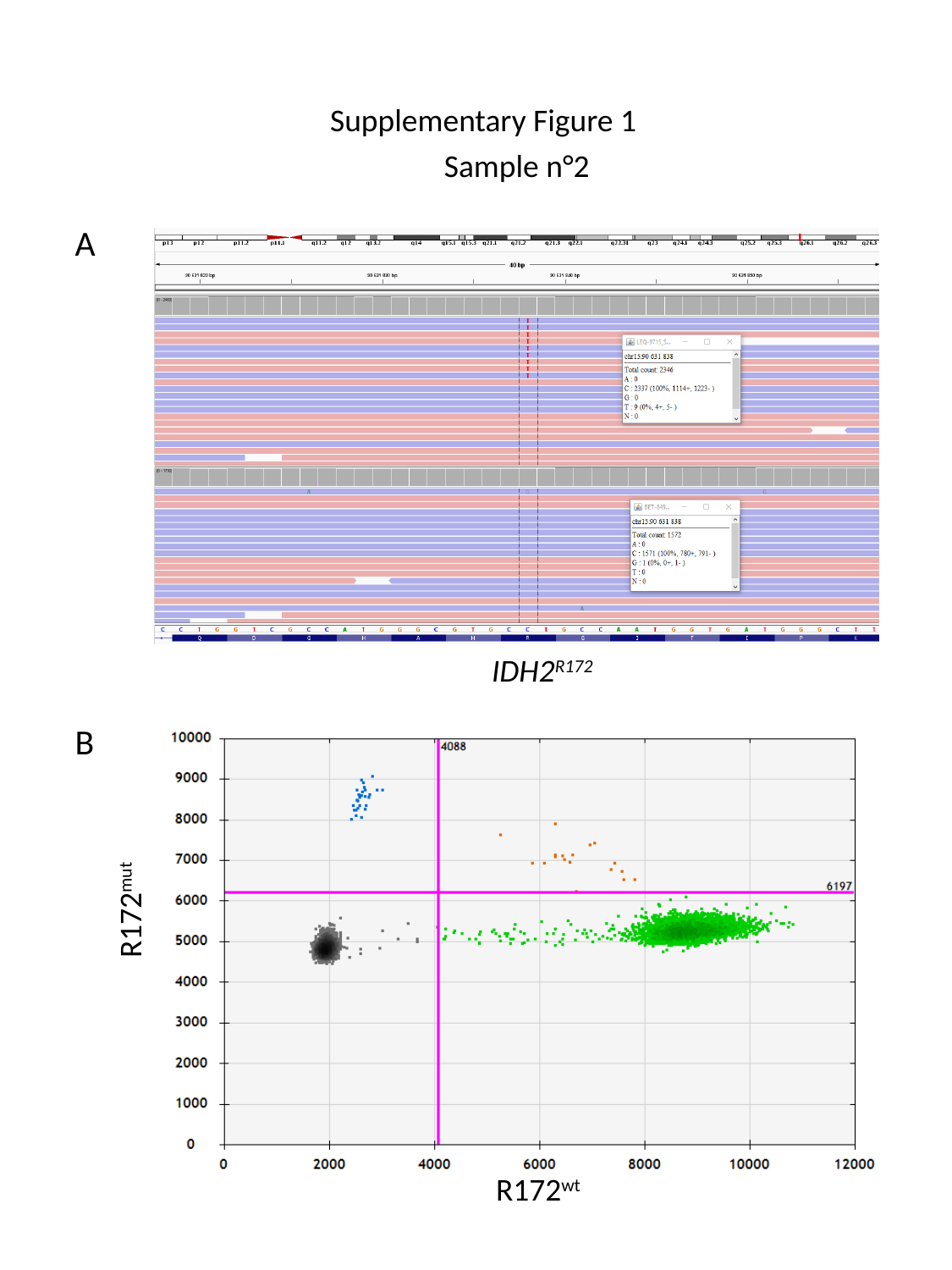

Supplementary Figure 1
Sample n°2
A
IDH2R172
B
R172mut
R172wt

Supplement: Supplementary file 2 — Figure S1. Detection of an additional mutation by ddPCR below the detection limit of NGS. [file MOL2-16-3916-s002.pptx]
